# Latex Hypersensitivity among Allergic Egyptian Children: Relation to Parental/Self Reports

**DOI:** 10.1155/2014/629187

**Published:** 2014-11-17

**Authors:** Zeinab A. El-Sayed, Shereen S. El-Sayed, Rehab M. Zaki, Mervat A. Salama

**Affiliations:** Pediatric Allergy and Immunology Unit, Children's Hospital, Faculty of Medicine, Ain Shams University, Cairo, Egypt

## Abstract

*Background*. Latex allergy is one of the major health concerns and allergic reactions to latex may be serious and fatal. *Purpose*. In this study, we sought to determine the frequency of latex hypersensitivity in a group of allergic Egyptian infants and children and its relation to the history provided by the patients or caregivers. *Methods*. We consecutively enrolled 400 patients with physician diagnosed allergic diseases. The study measurements included clinical evaluation for the site and duration of allergy, history suggestive of latex allergy, family history of allergy, and skin prick testing (SPT) using a commercial latex extract. *Results*. The study revealed that 16/400 (4%) patients had positive SPT; 11 of them only had positive history of sensitivity to latex. Positive latex SPT was reported in 3.4% (11/326) of patients with bronchial asthma, 5.9% (7/118) of patients with skin allergy, and 4.5% (2/44) of patients with allergic rhinitis. SPT was positive in 7.4% (4/54) of patients with concomitant respiratory and skin allergy. Latex SPT was more specific than sensitive (97.69% and 77.77%, resp.) with a negative predictive value of 99.47%. *Conclusion*. Although underrecognized, latex is an important allergen in the pediatric age group with a sensitization frequency of 4% among allergic children. It was observed to be especially associated with multiple allergic diseases coexisting in the same patient. Pediatric allergologists should educate their patients on latex allergy and encourage the use of latex-free products.

## 1. Introduction

Latex as found in nature is a milky sap-like fluid found in 10% of all flowering plants [1]. Natural rubber latex (NRL) extracted from* Hevea brasiliensis* tree has been widely used in the manufacturing of gloves, balloons, and parts of medical and dental equipment [2]. Among more than 200 polypeptides identified in NRL as potential allergens,* Hevea brasiliensis* 6 [Hev 6] and Hev b1-13 are recognized as the primary allergens by the International Union of Immunological Societies [3]. Latex elongation factor Hev d1 is the relevant allergen in patients with spina bifida. Prohevein (Hev B6) behaves as a major allergen, since it reacts to IgE in most of the sera of patients with latex allergy [4]. Exposure to latex via direct skin contact or inhalation of airborne allergens from powdered gloves poses the risk of sensitizing both clinicians and their patients. The risk of developing latex hypersensitivity increases with prolonged and repeated exposure [5].

Latex sensitization is defined as the presence of immunoglobulin antibodies to NRL products without clinical manifestations [6]. Sensitization does not always lead to allergy. It remains unclear why someone who is exposed to latex does not develop a latex sensitivity whereas others who do develop this sensitivity do not manifest reactions on contact [7]. Sensitization rates to latex differ in various populations, ranging from 0.1% to 1% in the normal population and 2.6% to 16.9% in health care workers to 28% to 67% in patients with spina bifida [8].

The association of latex allergy and allergy to plant-derived foods is called latex-fruit syndrome and is attributed to the cross-reactivity between the major latex allergen hevein and hevein-like domains (HLDs) from fruit class 1 [9]. In patients with a history suggestive of latex sensitization, physicians should ask about skin and respiratory symptoms, as well as food allergies, particularly in patients with a history of atopy [10]. The skin prick test is the cheapest, practical, and the most widely used method for the diagnosis of allergic diseases [11]. The authors are not aware of previous work addressing the prevalence of skin sensitization to latex in allergic Egyptian children. Hence, this study was carried out to determine the prevalence of latex hypersensitivity among allergic children and its relation to the history provided by the patients or their caregivers.

## 2. Methods

### 2.1. Study Population

This study comprised 400 allergic infants and children enrolled consecutively from the Pediatric Allergy and Immunology Clinic, Children's Hospital, Ain Shams University, and the outpatient clinics of El-Mounira and El-Zawya hospitals over the period from March 2011 to June 2013. Patients included suffered clinical allergic disorders including bronchial asthma (BA), skin allergy, and allergic rhinitis (AR). An informed consent was obtained from the parents and caregivers prior to enrollment. Approval of the local ethics committee was obtained.

The diagnosis of BA was established according to the criteria of the American Thoracic Society [12]. The diagnosis of AR was established according to the guidelines of AR and its impact on asthma (ARIA) [13]. The diagnosis of atopic dermatitis was established according to the scoring atopic dermatitis index (SCORAD) [14] and the diagnosis of urticaria was established according to the criteria proposed by Zuberbier and Maurer [15].

### 2.2. Study Measurements

All patients included in the study were subjected to the following.

*(1) Clinical Evaluation*. Clinical history was taken for the allergic disorder and its duration, sensitivity to latex, and exposure to latex containing products including gloves. History of allergy to fruits such as banana and kiwi was also sought. Inquiry was made about the risk factors such as neural tube defects, urogenital anomalies, repeated surgical manoeuvers, and hand dermatitis.

*(2) Skin Prick Testing*. Skin prick tests were performed for each patient using ammoniated allergen extract for latex (Allergopharma Joachim Ganzer D-21462 Reinbek, Germany), positive control (histamine hydrochloride 10 mg/mL), and negative saline control. The procedure was first explained to each patient and/or the caregiver then consent was obtained. First generation short-acting anti-histamines were avoided for at least 72 hours and second generation antihistamines were avoided for at least 5 days before testing. The test sites were marked and labeled at least 3 cm apart, then dropped by the allergen, and gently pricked by sterile skin test lancet. Positive and negative control solutions were similarly applied. Epinephrine was ready in case any systemic reaction occurred. The test result was interpreted after 20 minutes. Largest and orthogonal diameters of any resultant wheal and flare were measured. Pseudopod formation was considered a significant positive reaction. A wheal of 3 mm or more above the negative control was taken as a positive result.

*(3) Laboratory Investigations*. Serum total IgE was measured by quantitative enzyme linked immunosorbent assay (ELISA) (Medix Biotech, Inc., Agenzyme Company, Industrial Road, San Carlos, CA, USA). Due to variations of serum IgE with age, the patient's serum IgE value used for data analysis was calculated as a percentage of the highest normal for age. Complete blood counts were done on Coulter Counter (Coulter Microdiff 18, Fullerton, CA, USA).

### 2.3. Statistical Methods

SPSS for Windows, release 15.0 (SPSS Inc., USA), was used for data entry and analysis. All numeric variables were expressed as mean ± standard deviation (SD) or median (interquartile range (IQR)) as appropriate. Comparison of continuous variables was done using Student's *t*-test for normally distributed variables and Mann-Whitney test for nonparametric variables. Chi-square (*χ* ^2^) and Fisher exact tests were used for categorical variables as appropriate. Spearman's correlation test was used. For all tests a probability (*P*) less than 0.05 was considered significant.

## 3. Results

The study sample comprised 212 males (53%) and 188 females (47%) (Table 1). A positive family history of allergy was found in the first and/or second degree relatives of 269 (67.2%) of the subjects. Isolated respiratory allergy was present in 282 (70.5%) of our patients; 64 (16%) suffered from isolated skin allergy, while 54 (13.5%) had both skin and respiratory allergies. AR was present in 44 patients (11%), BA in 326 (81.5%), and skin allergy in 118 (29.5%) (N.B. some cases had more than one allergic condition).

History of exposure to balloons and rubber toys was positive in 358 (89.5%) patients, to latex bottle nipples in 166 (41.5%), to erasers in 203 (50.7%), and to latex gloves in 105 (26.2%). A history of allergy to fruits, namely, banana, kiwi, and pears, was found in 225 patients (56.25%). The presence of risk factors such as neural tube defect and urogenital anomalies with repeated urinary catheterization was found in 3 (0.75%) and hand dermatitis in 6 (1.5%).

### 3.1. Results of Latex SPT

**Figure 1:**
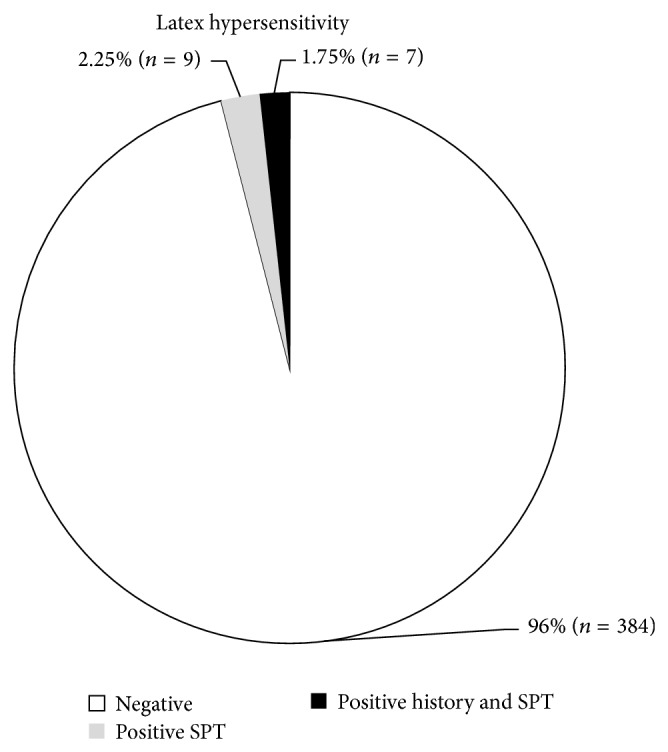
Latex sensitivity and SPT results.

The result was positive in 16/400 patients (4% of the studied sample). Seven of our patients (1.75%) had positive latex allergy; that is, they had positive history of allergy to latex products concomitant with positive latex SPT (Figure 1). Among those with latex positive SPT, 11/16 had positive family history of allergy and only one patient suffered from urogenital anomalies.

Positive history of latex allergy was significantly higher among patients with positive latex SPT having a frequency of 77.8% in comparison to 22.2% in those with negative latex SPT. The odds ratio (OR) was 148 (confidence interval: 27-817). Allergy to fruits was present in 7/16 (43.7%) of the patients who reacted positively to latex SPT versus 218/384 (56.7%) of those with negative result (OR (CI): 0.592 (0.216–1.62); insignificant).

### 3.2. The Results of Latex SPT according to the Type of Allergic Disease

Positive SPT to latex was observed in only 3.2% (9/282) of patients with isolated respiratory allergy and 4.7% (3/64) of patients with isolated skin allergy in contrast to 7.4% (4/54) of patients with concomitant respiratory and skin allergy (*X* ^2^ = 2.19; *P* = 0.334).

Positive SPT to latex was seen in 3.4% (11/326) of all patients with bronchial asthma, 5.9% (7/118) of patients with skin allergy (all the 7 had urticaria), and 4.5% (2/44) of patients with AR (OR (CI): 0.48 (0.16–1.4); 1.9 (0.6–5.2); 1.16 (0.25–5.2), resp.).

The patient's age, duration of exclusive breast feeding, age of weaning, duration of the allergic disease, absolute eosinophilic count, total serum IgE, and IgE percent did not bear a statistically significant relation with positivity of latex SPT (*P* = 0.33, 0.25, 0.98, 0.25, 0.92, 0.73, and 0.477, resp.).

The sensitivity of latex SPT was 77.77% and specificity was 97.69% with positive predictive value of 43.75% and negative predictive value of 99.47% with an overall efficacy of 97.25%.

## 4. Discussion

Immediate hypersensitivity to natural rubber latex has increased since the early 1980s. High prevalence of latex sensitization and allergy are observed among health care workers, atopic individuals, and children who are exposed to multiple surgical maneuvers such as spina bifida and urogenital anomalies [16].

In children, latex sensitization prevalence studies are scarce and involve different population sampling and allergy testing methods, which makes it difficult to compare across studies. Aiming at determining the brunt of the problem in allergic children and its relation to positive history as reported by the patients or their caregivers, we studied the prevalence of latex sensitization in a sample of allergic Egyptian children. This might help in deciding the need for measures to reduce the problem.

In the present study, latex SPT revealed that 4% of the studied sample (16/400) was sensitized to latex. There are no reports on the size of the latex sensitization problem in Egypt. A positive history of exposure to latex products was highly encountered in our cohort of allergic children reaching as high as 89.5% for balloons and rubber toys and 26.2% for latex gloves. Latex containing products such as toys, bottle nipples, and erasers are widely used in Egypt. It is to be noted that most hospitals in Egypt are still using latex-containing medical gloves. However, few dentists and physicians became aware of this problem and started introduction of latex-free medical gloves in their private clinics. Moreover, latex allergens present in sediment and airborne particulate material, derived from tire debris due to heavy urban vehicle traffic and from latex industries in our country (such as latex paints, mattresses, and medical instruments), could be important factors in producing latex sensitization. In a study of 326 atopic children, 10 (3%) presented positive skin test to latex, but only five (1.5%) also had a positive clinical history to latex exposure [17]. The prevalence of sensitization to latex was 9% in atopic Danish children, but the prevalence of manifest type 1 latex allergy was only 1% [18]. A frequency of 4.3% was reported among 2352 Japanese children under 14 years of age with different allergic diseases [19].

Meglio and associates [20] studied a sample of 151 atopic Italian children and they found that 6 patients (3.9%) had positive SPT to latex. A lower prevalence was reported by Nettis and coworkers [21] who found that 2.8% of 1000 atopic Italian patients had latex sensitization.

The corresponding percentage in the general pediatric population is 0.3–4% as reported by some authors [6]. Moreover, Jorge and colleagues [22] studied 182 children from the outpatient clinics of two different hospitals and found that 3.8% had positive latex SPT. However, Roberts and coworkers [23] studied a sample of 1877 children at 7 years of age in the United Kingdom and they found that only 0.2% of the study population had positive latex SPT.

In the present study, 7/16 of those with positive latex SPT gave positive history of allergy to latex. This was a significant finding as compared to those with negative SPT, with a high odds ratio OR (CI): 148 (27–817). The latex SPT was specific rather than sensitive (97.6% versus 77.7%) and showed a high negative predictive value of 99.47%. In an earlier study, the sensitivity of latex SPT was 100% and the specificity was 98% [20]. In children with clinically confirmed latex allergy, sensitivity and specificity of different commercially available skin prick tests could vary. Ammoniated latex extract has shown a higher sensitivity in comparison with nonammoniated products [24].

The frequency of latex sensitization in all our asthmatic patients was 3.4% (11/326). In a sample of 1097 patients with occupational asthma, 4.9% were found sensitized to latex [25]. A higher prevalence rate of latex sensitization of 10% was recorded in adult asthmatics [26]. Sensitization to cockroach and latex was rare among Danish children with verified asthma [27].

In our series, 2/44 (4.5%) AR patients had positive latex SPT. In comparison, Airaksinen and colleagues [28] reported that 10% of the 829 individuals with suspected occupational rhinitis had positive inhalation challenge test to latex. Also, Kimata [19] studied 802 children with AR in three consecutive years between 2001 and 2003 and found that the prevalence of latex allergy was 3.1/5.1/9.1%, respectively.

Our study population included 118 patients with skin allergy (+/− other allergic diseases) where 7 of them (5.9%) had positive SPT to latex. The incidence of latex allergy among 844 patients under 14 years of age with skin allergy in 2001/2002/2003 was 6.1/11.3/15.9%, respectively, denoting a steady rise [19]. Worth mentioning is that among our 118 patients with skin allergy 101 (85.5%) had urticaria, whereas in the other studies all the patients had AEDS. Patients with contact urticaria were reported to have a significantly poorer prognosis than those with contact allergy [29]. In an Egyptian study, latex specific IgE was significantly high in asthmatic children (*n* = 22) (mean ± standard deviation: 2.09 ± 6.39) but not in those with atopic dermatitis (*n* = 8) (0.09 ± 0.16) as compared to controls [30].

The coexistence of more than one allergic disease in the same patient might increase the possibility of having latex hypersensitivity based on the finding in the present work that 7.4% (4/54) of patients with both respiratory and skin allergy had positive latex SPT in contrast to only 3.2% of patients with respiratory allergy and 4.7% of those with skin allergy.

Our study population included 3 patients with neural tube defects and urogenital anomalies necessitating frequent exposure to latex made catheters. Latex skin prick test was positive in one (25%). Spartà and associates [31] studied 85 children with urogenital defects with a median age of 10.5 years and found that 11 (12.9%) of them had positive specific IgE. In the study of El-Sayed and associates [30], latex specific IgE (mean ± standard deviation) was significantly high in children with repeated instrumentation (*n* = 17) (2.89 ± 3.66) with a frequency 52% of latex sensitization among children with spina bifida and urogenital anomalies as denoted by latex specific IgE. In Brazil, a prevalence of 25% for latex sensitization and of 20% for latex allergy was reported among 55 studied patients with meningomyelocele [32].

In the present study, half of all allergic children had history of fruit allergy (43.7% of patients with positive and 56.7% of those with negative latex SPT). Only 6 out of 222 patients with history of banana allergy (2.7%) had positive latex SPT and only 2/6 had positive history of latex allergy. The only patient who had history of kiwi allergy had positive latex SPT and denied history of latex allergy. Overall, the difference was insignificant (OR (CI): 0.59 (0.2–1.62)) perhaps indicating that history of fruit allergy should be confirmed by SPT or oral challenge before considering it as a risk factor for latex allergy. Other fruits that cross-react with latex such as avocado are not popular in Egypt. Radauer et al. [9] also found no significant correlation between latex associated plant food allergy and sensitization to hevein and HLDs which are major latex allergens.

It is concluded that latex skin sensitization was found in 4% of the studied allergic children, yet latex allergy as determined by a positive self/parental report and positive skin prick test was observed in only 1.75%. Although underrecognized, latex is an important allergen in the pediatric age group. It was observed to be especially associated with multiple allergic diseases coexisting in the same patient. Pediatric allergologists should educate their patients on latex allergy and encourage the use of latex-free products. Studies on the prevalence of latex sensitization in the general population as well as studies on environmental air pollution with latex are recommended.

## Figures and Tables

**Table 1:** Demographic and clinical data of patients.

Parameter		Positive latex SPT *N* = 16 (4%)	Negative latex SPT *N* = 384 (96%)	*P* value
Sex	Male	8 (2%)	204 (51%)	0.806
	Female	8 (2%)	180 (45%)	

Age (years)	Range	1–17	0.5–15	0.338
	Median	5.2	4.5	
	Interquartile range (IQR)	9.7	4	

Duration of exclusive breast feeding (months)	Mean	5.33	5.7	0.257
	SD	1.29	1.4	

Site of allergy	Respiratory allergy only (BA, AR, or both)	9 (2.25%)	273 (68.25%)	0.334
	Skin allergy only	3 (0.75%)	61 (15.25%)	
	Both respiratory and skin	4 (1%)	50 (12.5%)	

Diagnosis	All BA cases	11 (2.75%)	315 (78.75%)	0.18
	All AR cases	2 (0.5%)	42 (10.5%)	0.845
	All skin allergy cases	7 (1.75%)	111 (27.75%)	0.202

Duration of illness (years)	Median (IQR)	3.25 (3)	2.5 (4)	0.251

History of latex allergy		7 (1.75%)	2 (0.5%)	0.000

Exposure to latex gloves		8 (2%)	97 (24.25%)	0.104

Positive family history of allergy		11 (2.75%)	258 (64.5%)	0.896

Fruit allergy		7 (1.75%)	218 (54.5%)	0.304

Absolute eosinophilic count ×10^3^/Cu*·*mm	Median	0.2	0.2	0.928
	IQR	0.27	0.3	

IgE% of normal for age	Median	96.3	66.67	0.477
	IQR	133.3	105.53	

BA: bronchial asthma.

AR: allergic rhinitis.
